# Effect of Fe_3_O_4_ Nanoparticles on Mixed POPC/DPPC Monolayers at Air-Water Interface

**DOI:** 10.1155/2019/5712937

**Published:** 2019-03-03

**Authors:** Zhuangwei Xu, Changchun Hao, Bin Xie, Runguang Sun

**Affiliations:** School of Physics and Information Technology, Shaanxi Normal University, Xi'an 710062, China

## Abstract

Fe_3_O_4_ nanoparticles (NPs) as a commonly used carrier in targeted drug delivery are widely used to carry drugs for the treatment of diseases. However, the mechanism of action of between Fe_3_O_4_ NPs and biological membranes is still unclear. Therefore, this article reports the influence of hydrophilic and hydrophobic Fe_3_O_4_ NPs on mixed 1-palmitoyl-2-oleoyl-*sn*-glycero-3-phosphocholine (POPC) and 1,2-dipalmitoyl-*sn*-glycero-3-phosphocholine (DPPC) that were studied using the Langmuir-Blodgett (LB) film technique and an atomic force microscope (AFM). From surface pressure-area (*π*-*A*) isotherms, we have calculated the compression modulus. The results showed that hydrophobic Fe_3_O_4_ NPs enlarged the liquid-expanded (LE) and liquid-condensed (LC) phase of the mixed POPC/DPPC monolayers. The compressibility modulus of the mixed POPC/DPPC monolayer increases for hydrophilic Fe_3_O_4_ NPs, but the opposite happens for the hydrophobic Fe_3_O_4_ NPs. The adsorption of hydrophobic Fe_3_O_4_ NPs in mixed POPC/DPPC monolayers was much more than the hydrophilic Fe_3_O_4_ NPs. The interaction of hydrophilic Fe_3_O_4_ NPs with the head polar group of the mixed lipids increased the attraction force among the molecules, while the interaction of hydrophobic Fe_3_O_4_ NPs with the tail chain of the mixed lipids enhanced the repulsive force. The morphology of the monolayers was observed by AFM for validating the inferred results. This study is of great help for the application of Fe_3_O_4_ NPs in biological systems.

## 1. Introduction

Nowadays, nanoparticles (NPs) have been used in the research of new materials, biological imaging, biosensors, drug delivery, and other biotechnologies or biologically related systems [1–5]. Fe_3_O_4_ NPs, which have the characteristics of low toxicity and high biocompatibility, especially play a unique role in the study of drug delivery systems [6–9]. Based on the magnetic properties of Fe_3_O_4_ NPs, it can be used as a carrier to carry targeted drugs to be accurately transported to cancer cell areas. However, in order for nanoparticles to enter the cell, it is necessary to understand the interaction of the nanoparticles with the biofilm. Hence, it is urgent to study the effects of Fe_3_O_4_ NPs and biofilms.

A biological membrane mainly contains all kinds of lipids, cholesterols, and proteins [10, 11]. Owing to the complexity of its composition, researches have used the model of lipids to study its structural characteristics. In the past years, people mainly studied the effects of Fe_3_O_4_ on the single lipid layer [1]. There were few researches who committed to Fe_3_O_4_ NPs and multilipids, especially to compare the different properties of Fe_3_O_4_ NPs with those of biofilm models. Therefore, it is necessary to study the effects of hydrophilic and hydrophobic Fe_3_O_4_ NPs on mixed lipid monolayers.

The Langmuir-Blodgett (LB) method is one of the most favorable tools for the in vitro study of the interaction at the air-water interface [12–14]. The importance of electron microscopy to modern technology is self-evident, and the atomic force microscope (AFM) is a scanning probe microscope known for its unique measurement conditions (room temperature and no vacuum) and high-resolution surface topography. AFM is widely used in many fields, such as in materials science, nanotechnology, biology, and the semiconductor industry [15]. Due to the nanoscale of the AFM tip, it can better observe the surface topography and structure of the nanoparticles and biofilm simulation. In this study, we used 1-palmitoyl-2-oleoyl-*sn*-glycero-3-phosphocholine (POPC) and 1,2-dipalmitoyl-*sn*-glycero-3-phosphocholine (DPPC) lipids as binary biological membrane models to explore the interaction of hydrophilic and hydrophobic Fe_3_O_4_ NPs with biological membrane models. We use LB and AFM techniques to study the stability, fluidity, and adsorption of monolayers.

## 2. Materials and Methods

### 2.1. Materials

1,2-Dipalmitoyl-*sn*-glycero-3-phosphocholine (DPPC) and 1-palmitoyl-2-oleoyl-*sn*-glycero-3-phosphocholine (POPC) were purchased as powders from Avanti Polar Lipids (AL, USA). Hydrophilic Fe_3_O_4_ NP solution (10 nm avg. part. size, 5 mg/mL in H_2_O) and hydrophobic Fe_3_O_4_ NP solution (10 nm avg. part. size, 5 mg/mL in toluene) were purchased from Sigma-Aldrich. In these experiments, the water was the Milli-Q water (18.2 MΩ·cm) obtained from a Millipore purification system.

### 2.2. Methods

Monolayer experiments were carried out with Langmuir-Blodgett (LB) films (KSV Minitrough, Finland). These are made up of two barriers and a Wilhelmy plate. In the experiments, a Langmuir trough and two barriers were cleaned at least three times with anhydrous ethanol and ultrapure water alternately. The temperature of the subphase was maintained at 22 ± 0.5°C by circulating water equipment. The exact volume of the lipid solution was added to the air-water interface by a Hamilton microsyringe [16].

The surface pressure-area (*π*-*A*) isotherm curve of the monolayer can be autoobtained by computer. In order to increase the reliability of the experimental data, the experimental data were repeatedly computed at least three times. The monolayer was deposited onto freshly cleaved mica at the surface pressures of 5 mN/m and 20 mN/m by a pulling device with a speed of 1 mm/min. The transfer ratio is close to a unit, indicating that mica is almost completely covered with single layer.

The microstructures of LB monolayers were observed using the SPM-9500-J3 atomic force microscope (AFM) (Shimadzu Corp., Japan) in tapping mode at room temperature. The AFM images of the maximum scanning area of 125 × 125 *μ*m and a *Z* range of about 8 *μ*m was collected using a micro-V-shaped cantilever probe (Olympus Optical Co. Ltd., Japan). The probe was made of Si_3_N_4_ with a spring constant of 0.06 N/m and a tip radius of 10 nm. The images were collected simultaneously with 512 × 512 points and a scanning rate of 1.0 Hz per line.

## 3. Results and Discussion

### 3.1. *π*-*A* Isotherms and *C*_*s*_^−1^ of Monolayers with Hydrophilic and Hydrophobic Fe_3_O_4_ NPs

**Figure 1:**
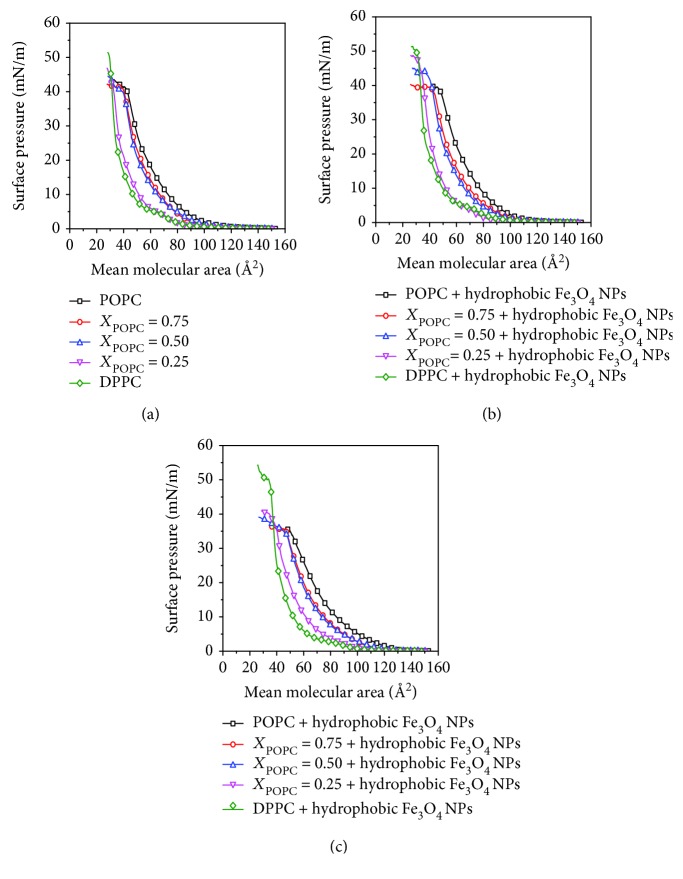
The surface pressure-area (*π*-*A*) isotherms of a mixed POPC/DPPC monolayer. (a) Pure lipids; (b) hydrophilic Fe_3_O_4_ NPs; (c) hydrophobic Fe_3_O_4_ NPs.

The surface pressure-area (*π*-*A*) isotherms can be used to reflect the phase behavior and thermodynamic properties of the lipid monolayers. The *π*-*A* isotherms of POPC/DPPC monolayers with different molar ratios at the air-water interface are shown in Figure 1(a). For a pure DPPC monolayer, the coexistence region of the liquid-expanded and liquid-condensed (LE-LC) phase was observed at the surface pressure of 4.5 mN/m, which is consistent with literature [17]. When *X*_POPC_ = 0.25, the phase transition point of the LE-LC phase was still observed. However, when *X*_POPC_ was increased to 0.5 and 0.75, the plateau-like LE-LC phase disappeared. The phase transition temperatures of DPPC and POPC are 41°C and -2°C, respectively [18]. At room temperature, the DPPC phase is in the gel phase and the POPC phase is in the liquid phase. The mixture of POPC/DPPC showed a different phase behavior from pure lipids.

Figures 1(b) and 1(c) show the *π*-*A* isotherms of mixed POPC/DPPC monolayers with the different subphase, respectively. In the subphase, the concentration of hydrophilic and hydrophobic Fe_3_O_4_ NPs was 0.016 mM, which was consistent in all experiments. We found that the isotherms moved to the direction of a larger area for the hydrophobic Fe_3_O_4_ NP case than for the hydrophilic Fe_3_O_4_ NP case.

**Figure 2:**
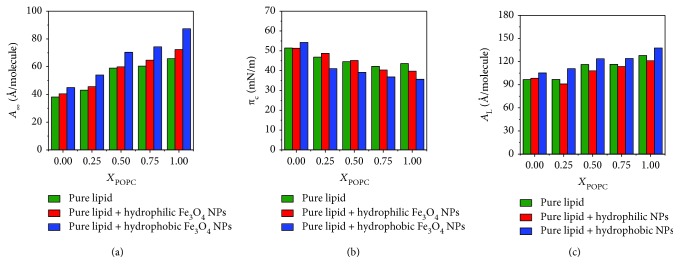
(a) Limiting area *A*_∞_. (b) Collapse pressure *π*_c_. (c) Lift-off area *A*_L_ of mixed POPC/DPPC monolayers with different subphases.

In order to more easily understand the effect of hydrophilic/hydrophobic Fe_3_O_4_ NPs on the isotherms of the lipid monolayers, the relevant information is summarized in Figure 2. The lipid monolayers have three characteristic parameters: limiting area *A*_∞_ (an empirical parameter approximating the area occupied by the molecules at zero pressure), collapse pressure *π*_c_, and lift-off area *A*_L_ (the molecular occupation area where the isotherm rising just emerges related to the baseline) [19].

The limiting area of pure DPPC monolayers is only 38.8 Å, while the limiting area of pure POPC is 65.76 Å. After the hydrophilic/hydrophobic Fe_3_O_4_ NPs are added to the subphase, the limiting area of the monolayers increases and the hydrophobic Fe_3_O_4_ NPs are larger than the hydrophilic Fe_3_O_4_ NPs. The collapse pressure decreases with the increase of *X*_POPC_. In the presence of POPC molecules, hydrophobic Fe_3_O_4_ NPs significantly affect the collapse pressure of the monolayers, resulting in a significant dropping of the collapse pressure. Compared with pure DPPC monolayers, the lift-off area of DPPC monolayers is higher in the presence of hydrophilic Fe_3_O_4_ NPs, but lower in comparison with hydrophobic Fe_3_O_4_ NPs.

The compression modulus *C*_*s*_^−1^ can be calculated from the *π*-*A* isotherms [20, 21] to study the compression or elastic properties of the Langmuir monolayers. It is calculated as follows:

(1) $$\begin{array}{c} C_{s}^{-1}=-A{\left(\frac{\partial \pi }{\partial A}\right)}_{T}, \end{array}$$

where *A* and *π* are the mean molecular area and surface pressure, respectively.

**Figure 3:**
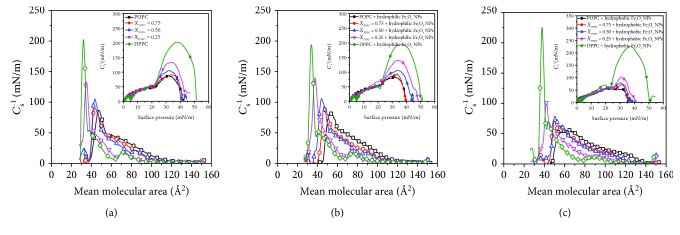
The compression modulus versus area (*C*_*s*_^−1^ vs. *A*) of mixed POPC/DPPC (pure POPC black, DPPC olive, *X*_POPC_ = 0.75 red, *X*_POPC_ = 0.5 blue, and *X*_POPC_ = 0.25 magenta) with different subphases. (a) Water; (b) hydrophilic Fe_3_O_4_ NPs; (c) hydrophobic Fe_3_O_4_ NPs. Inset: plot of *C*_*s*_^−1^ vs. *π* dependencies.

The maximum value of compression modulus *C*_*s* max_^−1^ indicates the rigid state of Langmuir monolayers.  Figure 3 represents the compression modulus versus area (*C*_*s*_^−1^ vs. *A*) of certain molar ratios of mixed POPC/DPPC (pure POPC black, DPPC olive, *X* = 0.75 red, *X* = 0.5 blue, *X* = 0.25 magenta) at the air-water interface.

**Figure 4:**
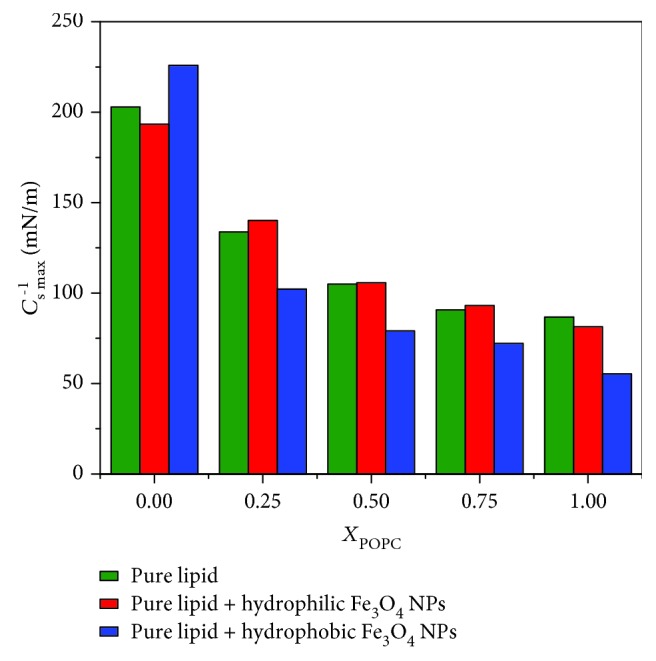
Compression modulus *C*_*s* max_^−1^ of mixed POPC/DPPC monolayers with different subphases.

For convenience, the maximum value of compression modulus *C*_*s* max_^−1^ is shown in Figure 4. In Figure 4, the *C*_*s* max_^−1^ values of pure DPPC and POPC monolayers are 202.89 mN/m and 86.78 mN/m, respectively, indicating that they are in the liquid-gel phase and the liquid phase, respectively. As the proportion of POPC increases, the *C*_*s* max_^−1^ values of the mixed POPC/DPPC monolayers decrease, indicating that the phase transitions of mixed lipids are in the coexistence of the gel phase (DPPC) and liquid phase (POPC). The presence of POPC obviously enhances the fluidity of Langmuir monolayers. The compressive modulus of DPPC decreased slightly when hydrophilic Fe_3_O_4_ NPs were added into the subphase. However, the addition of hydrophobic Fe_3_O_4_ NPs can increase the rigidity of the DPPC monolayer.

### 3.2. The *π*-*t* Adsorption Curve of Mixed POPC/DPPC Monolayers

**Figure 5:**
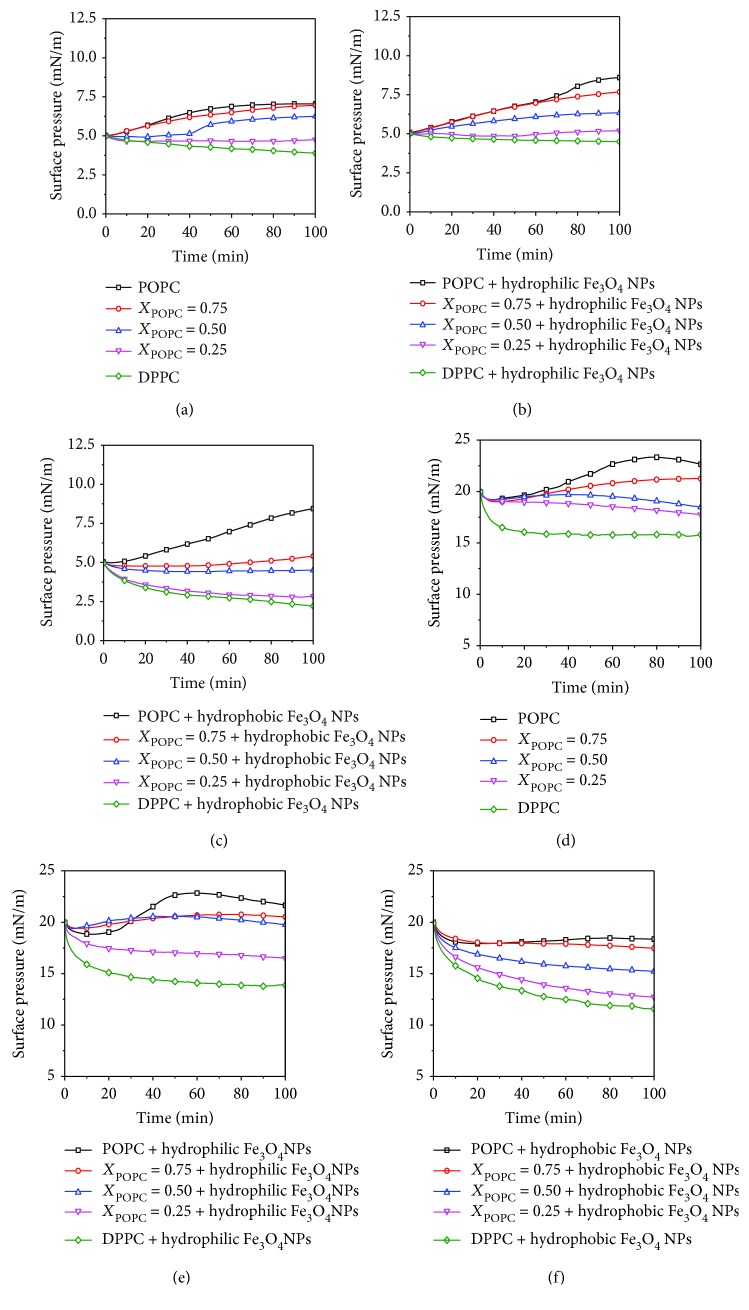
Surface pressure-time (*π*-*t*) adsorption curve of different molar ratios of mixed POPC/DPPC at the initial surface pressure of 5 mN/m and 20 mN/m. (a and d) Lipid, (b and e) hydrophilic Fe_3_O_4_ NPs, and (c and f) hydrophobic Fe_3_O_4_ NPs.

In our experiments, to understand the adsorption or permeability of exogenous substances such as drugs or nanoparticles to the cell membrane, the change of adsorption capacity is usually explained by the change of surface pressure over time [1, 22].  Figure 5 shows the surface pressure-time (*π*-*t*) adsorption curve of different molar ratios of mixed POPC/DPPC at the initial pressure of 5 mN/m and 20 mN/m.  Figure 5(a) shows the change of surface pressure of the DPPC and POPC monolayers at an initial surface pressure of 5 mN/m. The surface pressure of the DPPC monolayer gradually decreases with increasing time. The surface pressure of the mixed lipid monolayers increases slightly as the proportion of POPC increases. We found that the adsorption curves of the mixed POPC/DPPC with hydrophilic/hydrophobic Fe_3_O_4_ NPs were different from the pure lipids (Figures 5(b) and 5(c)). The surface pressure of mixed lipids in the presence of hydrophilic Fe_3_O_4_ NPs is higher than that of pure lipids, while the surface pressure of mixed lipids with hydrophobic Fe_3_O_4_ NPs is significantly lower than that of pure lipids. The change in the adsorption curves of both pure and mixed POPC/DPPC at an initial surface pressure of 20 mN/m is similar to the change in initial surface pressure of 5 mN/m, but the former is more varied than the latter. It is found from Figures 5(d) and 5(f) that the effect of hydrophilic and hydrophobic Fe_3_O_4_ NPs on the adsorption curve of mixed lipid monolayers is more notable at high surface pressure (20 mN/m).

At the initial surface pressure of 5 mN/m, the Δ*π* (surface pressure difference from 0 to 100 minutes) increases with the increases of *X*_POPC_. However, at the initial surface pressure of 20 mN/m, the Δ*π* decreases with the increase of *X*_POPC_. This may be due to the unsaturated tail chain of POPC molecules, which increases the fluidity of the monolayer and makes it easier for it to adsorb to the interface. It is also observed that hydrophobic Fe_3_O_4_ NPs have a greater influence on the interfacial adsorption capacity than hydrophilic Fe_3_O_4_ NPs. Due to the hydrophobic nature of nanoparticles, part of the lipid molecules are extruded from the interface or form NP-lipid complexes into the subphase. At the initial surface pressure of 20 mN/m, hydrophobic Fe_3_O_4_ NPs have a significant effect on the adsorption of lipid monolayers. The surface pressure of the adsorption curve have larger increases with the increase of *X*_POPC_, compared with that of DPPC monolayers. The change of high surface pressure is much more than that of low surface pressure. Because of the tight intermolecular arrangement of lipid monolayers at high surface pressures, the electrostatic repulsion of lipid tail chains is enhanced.

### 3.3. The Thermodynamic Analysis of Mixed POPC/DPPC Monolayers

For the miscibility and stability of mixed lipid monolayers, we can clarify their thermodynamic properties with the excess mean molecular area (*A*_exc_) and excess Gibbs free energy (Δ*G*_ex_). For binary mixtures, we give the surface pressure *π*, ideal area per molecule *A*_ideal_, excess mean molecular area *A*_exc__,_ and excess Gibbs free energy Δ*G*_ex_ [16, 23, 24]; they are defined as follows:

(2) $$\begin{array}{c} A_{\text{ideal }\left(\text{POPC}/\text{DPPC}\right)}=X_{\text{POPC}}A_{\text{POPC}}+X_{\text{DPPC}}A_{\text{DPPC}},\\ A_{\text{exc }\left(\text{POPC}/\text{DPPC}\right)}=A_{12}-A_{\text{ideal }\left(\text{POPC}/\text{DPPC}\right)},\\ \Delta G_{\text{ex}}=N_{A}\int_{0}^{\pi }A_{\text{exc}}d\pi , \end{array}$$

where *A*_POPC_ and *A*_DPPC_ are the areas per molecule of POPC and DPPC in pure monolayers at the considered *π*, and *X*_POPC_ and *X*_DPPC_ are the molar fractions of POPC and DPPC in the binary mixtures, respectively; *A*_12_ is the experimental evaluation of the area per molecule of the binary mixtures, *π* is surface pressure, and *N*_A_ is the Avogadro number.

**Figure 6:**
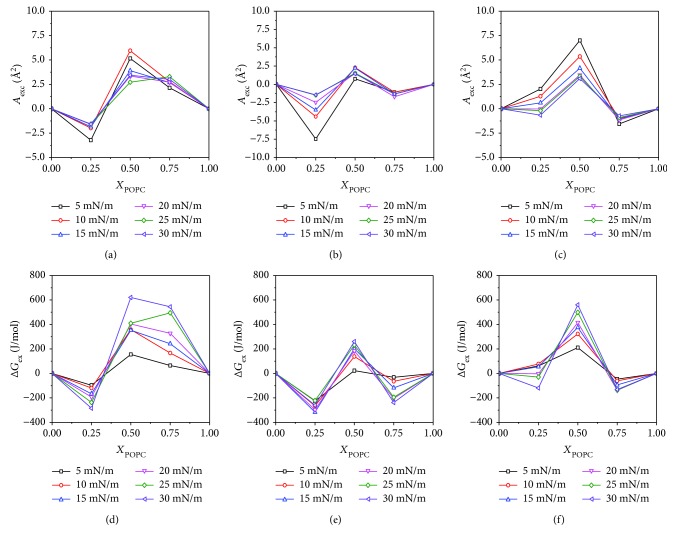
Excess mean molecular area (*A*_exc_) and excess Gibbs free energy (Δ*G*_ex_). (a and d) Lipid, (b and e) hydrophilic Fe_3_O_4_ NPs, and (c and f) hydrophobic Fe_3_O_4_ NPs.

The *A*_exc_ values of the mixed lipids in the case of ideal miscibility or complete immiscibility is zero [25]. According to previous reports, *A*_exc_ > 0 indicates that the lipid molecules are repulsive interactions, and *A*_exc_ < 0 indicates that the lipid molecules are attractive interactions [26].  Figure 6 shows the excess mean molecular area and excess Gibbs free energy of lipid monolayers. A negative value of *A*_exc_ (*X*_POPC_ = 0.25) indicates that there is an attractive interaction. A positive value of *A*_exc_ (*X*_POPC_ = 0.5) indicates that the equimolar POPC and DPPC have a repulsive interaction. In the experiments, hydrophilic Fe_3_O_4_ NPs enhanced the attraction interaction of mixed POPC/DPPC (*X*_POPC_ = 0.25) monolayers. The content of POPC molecules in the monolayers was further increased, and the molecular interaction in the mixed monolayers were transformed from repulsive to attractive interactions (Figure 6(b)). This is because the interaction between hydrophilic Fe_3_O_4_ NPs and the polar head of mixed lipids weakens the interaction of lipid molecules. In the presence of hydrophobic Fe_3_O_4_ NPs (Figure 6(c)), the value of *A*_exc_ (*X*_POPC_ = 0.25) decreases to the negative value as the surface pressure increases, indicating that the interaction between the force changes from repulsive force to attractive force. At *X*_POPC_ = 0.5 and *X*_POPC_ = 0.75, the values of *A*_exc_ are positive and negative, respectively. In contrast to hydrophilic Fe_3_O_4_ NPs, the repulsion interaction between lipid molecules is enhanced or the attraction interaction is attenuated in the presence of hydrophobic Fe_3_O_4_ NPs. In Figure 6(d), the value of Δ*G*_ex_ has a minimum at *X*_POPC_ = 0.25, indicating that the monolayers are most stable at POPC : DPPC (1 : 3), and the case of hydrophilic Fe_3_O_4_ NPs is similar to this condition. However, for the hydrophobic Fe_3_O_4_ NP subphase, the most stable lipid monolayers are POPC : DPPC (3 : 1).

### 3.4. The AFM Images of Pure POPC and DPPC and Different Molar Ratios of Mixed POPC/DPPC Monolayers with Hydrophilic and Hydrophobic Fe_3_O_4_ NPs at the Air-Water Interface

**Figure 7:**
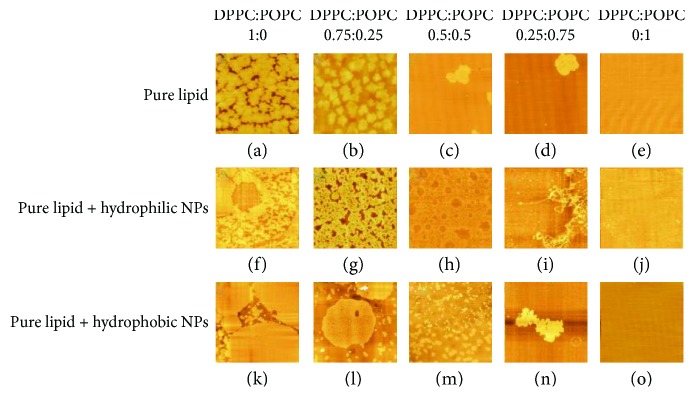
AFM images of mixed POPC/DPPC at the initial pressure of 5 mN/m.

**Figure 8:**
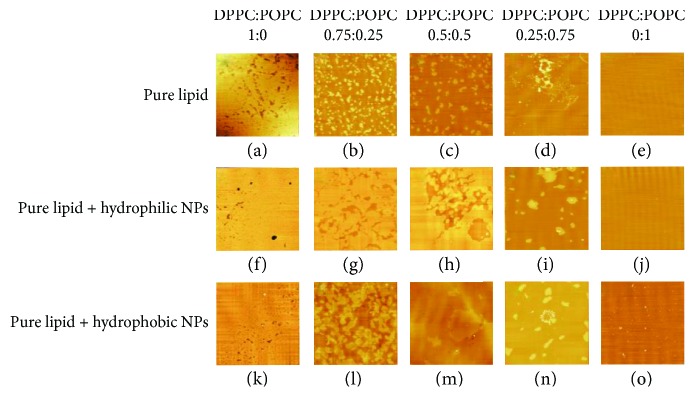
The AFM images of mixed POPC/DPPC monolayers at the initial pressure of 20 mN/m.

The AFM images of mixed POPC/DPPC monolayers at the initial pressures of 5 mN/m and 20 mN/m are shown in Figures 7 and 8, respectively.

In Figure 7(a), the pure DPPC monolayers at an initial surface pressure of 5 mN/m were in the LE-LC phase, and a polygonal irregular sheet-like structure was observed in the AFM image. With the increase of *X*_POPC_, it can be observed that the DPPC domains are gradually reduced and the POPC liquid phase appears. The AFM pattern of the DPPC monolayers with hydrophilic Fe_3_O_4_ NPs shows a partially dispersed platelet-like structure. The mixed POPC/DPPC (*X*_POPC_ = 0.25) monolayers with hydrophilic Fe_3_O_4_ NPs showed a porous, irregular sheet-like structure, which was more compact than pure *X*_POPC_ = 0.25. Due to the interaction between the hydrophilic Fe_3_O_4_NPs and the head of the DPPC molecules, the DPPC molecules or NP-DPPC complexes enter the subphase, which affects the DPPC monolayers in the interface arrangement. For the hydrophobic Fe_3_O_4_ NP condition, it showed many large patches of uniform structure. With the increase of *X*_POPC_, the large platform structure collapses into many small domains. This may be because of the repulsive interaction of hydrophobic Fe_3_O_4_ NPs with the tail chain of the mixed lipid molecules resulting in the lipid-NP complexes entering the subphase.

When the surface pressure is raised to 20 mN/m, the DPPC monolayers show a more uniform layered structure. With the increase of *X*_POPC_, the monolayer forms more small-area structures. The monolayers have the phase separation structure. At high surface pressure (20 mN/m), the lamellar structure of DPPC monolayers is more compact in the presence of hydrophilic Fe_3_O_4_ NPs. When the subphase is Fe_3_O_4_ NPs, the DPPC monolayers become more condensed. However, for the hydrophobic Fe_3_O_4_ NPs, the monolayer structure becomes more obvious. The interaction of hydrophilic Fe_3_O_4_ NPs with the lipid head enhances the attraction interaction between lipid molecules.

**Figure 9:**
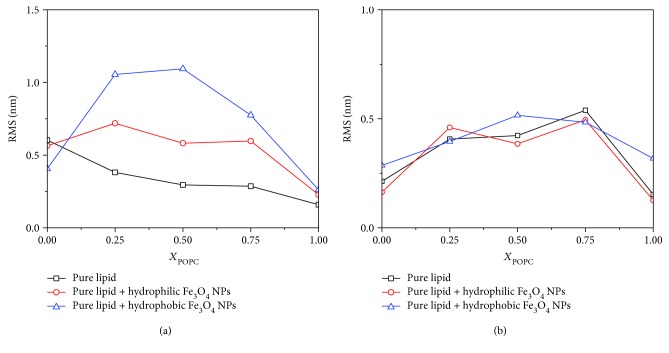
Surface roughness for the AFM images of mixed POPC/DPPC monolayers at the initial pressures of (a) 5 mN/m and (b) 20 mN/m.

Figures 9(a) and 9(b)) represent the surface roughness for the AFM images of mixed POPC/DPPC monolayers at the initial pressure of 5 mN/m and 20 mN/m, respectively.

In Figure 9, at an initial surface pressure of 5 mN/m, the presence of Fe_3_O_4_ NPs increases the surface roughness of the mixed POPC/DPPC monolayer, and the effect of hydrophobicity is stronger than that of hydrophilicity. However, when the initial surface pressure is 20 mN/m, the effect of Fe_3_O_4_ NPs on the roughness of mixed POPC/DPPC is not obvious, and the variation range is only 0.15 nm. This may be due to the disordered arrangement of lipid molecules at low surface pressure. When the surface pressure increases, the order between the lipid molecules increases and leads to the dense distribution of lipid molecules, and the effect of Fe_3_O_4_ NPs on the roughness of the mixed POPC/DPPC monolayer is not significant.

## 4. Conclusion

In this paper, the influence of the subphase of mixed POPC/DPPC monolayers was studied using the LB technique. The different content has great influence on the structure of monolayers. The subphase especially contains Fe_3_O_4_ NPs with different properties. To further illustrate the interaction of lipids with Fe_3_O_4_ NPs, we used AFM to study the surface morphology between them. The results show that the repulsive interaction of hydrophobic Fe_3_O_4_ NPs with the tail chain of the mixed lipid molecules results in the lipid-NP complexes entering the subphase. The interaction of hydrophilic Fe_3_O_4_ NPs with the lipid head enhances the attraction interaction between lipid molecules. Meanwhile, Fe_3_O_4_ NPs can increase the roughness of the mixed POPC/DPPC monolayer at low surface pressure and the effect of hydrophobicity is stronger than that of hydrophilicity; however, the effect is not obvious under high surface pressure. This study helps us gain new insights for the interaction between nanoparticles and molecules. This could have a potential application in designing the targeted drug liposomes.

## Data Availability

The data used to support the findings of this study are available from the corresponding author upon request.
